# Multidrug resistance and extended-spectrum beta-lactamase producing Gram-negative bacteria from three Referral Hospitals of Amhara region, Ethiopia

**DOI:** 10.1186/s12941-021-00422-1

**Published:** 2021-03-11

**Authors:** Feleke Moges, Mucheye Gizachew, Mulat Dagnew, Azanaw Amare, Bekele Sharew, Setegn Eshetie, Wondwossen Abebe, Yihenew Million, Tigist Feleke, Moges Tiruneh

**Affiliations:** 1 Department of Medical Microbiology, School of Biomedical and Laboratory Sciences, College of Medicine and Health Sciences, University of Gondar, Gondar, Ethiopia; 2 Department of Hospital Laboratory, University of Gondar Comprehensive Specialized Hospital, Gondar, Ethiopia; 3 Department of Medical Laboratory Sciences, College of Medicine and Health Sciences, Wollo University, Dessie, Ethiopia

**Keywords:** Gram-negative, Multi-drug resistance, Extended-spectrum beta-lactamase, Carbapenemase

## Abstract

**Background:**

Multidrug resistance (MDR), extended-spectrum beta-lactamase (ESBL) and carbapenemase-producing Gram-negative bacteria (GNB) has become a public health threat worldwide. This threat is worse in developing countries where there is high infectious disease burden and spread of antimicrobial resistance co-exist. The aim of the present study was, therefore, to assess MDR, ESBL and carbapenemase producing GNB from patients attending three selected referral hospitals in Amhara region.

**Methods:**

A cross-sectional study was conducted from December 2017- April 2018 at the University of Gondar Comprehensive Specialized Hospital, Dessie Referral Hospital and Debre Markos Referral Hospital of Amhara national regional state. A total of 833 study subjects were recruited using a convenient sampling technique. Clinical samples such as blood, urine, stool, wound, abscess, ear discharge, nasal discharge, cervical discharge and body fluid specimens were aseptically collected. Culturing for identification of bacteria and determination of drug susceptibility testing were done following standard microbiological techniques. Selected MDR isolates were phenotypically assessed for ESBL and carbapenemase production.

**Results:**

Of the 833 clinical samples cultured for bacterial growth, 141 (16.9%) were positive for GNB. The most common GNB identified were *E. coli* 46 (32.6%), *Klebsiella* spp. 38 (26.5%) and *Proteus* spp. 13 (9.2%). The overall MDR prevalence was 121 (85.8%). Among the total isolates, 137 (97.2%) were resistant to ampicillin followed by cotrimoxazole 115 (81.6%), amoxicillin-clavulanic acid 109 (77.3%), cefixime 99 (70.2%), cefepime 93 (66.0%) and tetracycline 91 (64.5%). The extended-spectrum beta-lactamase producing GNB were 69/124 (55.6%). Of which *Klebsiella* spp. 19 (15.3%) and *E. coli* 17 (13.7%) were common ESBL producers. Carbapenemase-producing isolates were 8/51(15.7%). Of which *Enterobacter*, *Klebsiella* and *E. coli* were common carbapenemase producers.

**Conclusion and recommendation:**

Multi-drug resistance and ESBL producing isolates in the present study were high. *E. coli* and *Klebsiella* spp*.* were the most common ESBL producing GNB. *Klebsiella* spp*., Enterobacter* spp*., E. coli* and *Citrobacter* spp*.* were typical carbapenemase-producing isolates. Continuous monitoring, antibiotic stewardship and molecular detection of the gene responsible for drug resistance are important means to reduce the spread of drug-resistant pathogens.

## Introduction

The rapid increase and spread of multidrug resistance (MDR) Gram-negative bacterial infections in hospitals and community has become one of the world’s greatest threats, due to the limited availability of alternative effective therapeutic options [1, 2]. The problem is even worse in developing countries where a high infectious disease burden and spread of antimicrobial resistance co-exists [3]. As a result, antimicrobial resistance is estimated to cause 700,000 deaths per year, a figure estimated to increase to 10 million deaths per year by 2050, as current therapies lose their effectiveness and antimicrobial-resistant infections spread [4]. The most commonly used classes of antimicrobial agents, for the treatment of infections caused by Gram-negative pathogens, include the fluoroquinolones, cephalosporins, and β-lactam/β-lactamase inhibitor combinations. Resistance to these agents would compromise the efficacy of empiric treatment of suspected Gram-negative infections [5].

The Beta-lactam antimicrobial agents are the most important drug of choice for the treatment of bacterial infections and remain to be the prominent source of resistance to Gram-negative bacteria worldwide. The persistent contact of bacterial strains to a multitude of β-lactams has induced an energetic and constant production and mutation of β-lactamases in these bacteria, escalating their activity even against the newly developed β-lactam antibiotics [6]. These enzymes are known as extended-spectrum β-lactamases (ESBLs) [7]. An overall pooled estimate of 42% ESBL was reported in east African hospitals [8]. Some reports from Ethiopia also showed that, there is a high prevalence of multidrug-resistant and ESBL-producing Gram-negative bacteria exist [9, 10]. However, the prevalence of ESBL production among clinical isolates varies from country to country and from institution to institution [6].

Carbapenem antibiotics are effective against multidrug-resistant Gram-negative bacteria, particularly those producing extended-spectrum β-lactamase, as well as a broad range of Gram-positive bacteria. However, their usefulness is threatened by the emergence and spread of bacteria that produce carbapenemase [11].

Carbapenemase-producing Gram-negative bacteria under the family Enterobacteriaceae are resistant to nearly all available antibiotics and are of particular concern in health care settings [12]. The dissemination of some multidrug-resistant isolates including carbapenemase producers occur rapidly. As a result, continuous monitoring, coordinated surveillance and control strategies at all levels of the health system should be undertaken [13]. Local epidemiological data along with local resistance patterns is fundamental for reducing mortality and morbidity of infections due to resistant bacteria. However, there are no comprehensive regional estimates of MDR, ESBL and carbapenemase-producing Gram-negative bacteria from different clinical samples. Therefore, the present study was aimed to determine the prevalence of multidrug resistance, ESBL and carbapenemase-production among the clinical isolates in the three referral hospitals of Amhara region, Ethiopia.

## Materials and methods

### Study design, period and area

A cross-sectional study was conducted from December 2017- April 2018 in the Amhara national regional state of the three referral hospitals. These are: University of Gondar Comprehensive Specialized Hospital (UGCSH), which is located in Gondar, 750 km northwest of Addis Ababa, and serves more than five million inhabitants in the Amhara region; Dessie Referral Hospital (DRH) which is a Zonal Hospital and serves three million people in South Wollo Zone; and Debre Markos Referral Hospital (DMRH) is also a Zonal Hospital, and serves two million people in East Gojjam Zone.

### Study populations, sample size and sampling technique

The source population was patients who were attending the University of Gondar Comprehensive Specialized Hospital, Dessie Referral Hospital and Debre Markos Referral Hospital seeking treatment during the study period. The study populations were all patients suspected of having bloodstream, UTI, wound and other infections. Patients who were on antibiotics treatment within the last two weeks before visiting the hospitals were excluded from the study. We have estimated the sample size using a single population proportion formula considering the previous study, P = 78.57% [14]; and a 5% margin of error. Therefore, considering a 5% non-response rate, the minimum sample size of the 3 referral hospitals together was 815. As a result, samples were collected from 833 patients by using convenient sampling technique. Written consents were obtained from study participants/ assents from parents or guardians. Patient’s sociodemographic characteristics were taken and clinical samples such as blood, urine, stool, wound, abscess, ear discharge, nasal discharge, cervical discharge and body fluid specimens were aseptically collected.

### Sample collection, processing and bacterial identification

Blood samples of 10 ml from adults, 5 ml from pediatrics age group and 2 ml from neonates were aseptically collected by using 70% alcohol and 2% iodine tincture (2 bottles for each patient at the different time points). The blood samples were inoculated simultaneously in tryptic soya broth (OXOID UK) and incubated immediately aerobically at 37˚C for five days and were checked for turbidity, hemolysis and clot formation daily. Bottles which showed signs of growth were further processed by Gram stain and sub-cultured on to blood agar, chocolate agar and MacConkey agar. The chocolate agar plates were incubated in a carbon dioxide atmosphere for up to 48 h, and the blood agar and MacConkey agar plates were incubated aerobically overnight. Those blood culture bottles which did not show growth were continuously monitored for the potential growth of pathogens for five days and if no growth was observed after five days, the blood culture was reported as negative [15].

Mid-stream urine samples were collected using a sterile test tube and inoculated on to Cysteine Lactose Electrolyte Deficient agar (CLED) using a calibrated loop (1.3 mm diameter, delivering1 μL) and incubated overnight at 37 °C. The samples with significant bacteriuria (≥ 10^5^ CFU/mL) were sub-cultured on to blood agar, MacConkey and Chocolate agar. Bacteria from other clinical samples such as stool, wound and other body fluids were processed following standard procedures [15].

Identification of Gram-negative bacteria was done using colony characteristics, Gram reaction and different biochemical tests such as, triple sugar iron agar, indole, motility, urease production, hydrogen sulphide production, citrate utilization, and lysine decarboxylase tests [15].

### Antibiotic susceptibility testing

Following identification of bacterial isolates, modified Kirby-Bauer disk diffusion method was done on Muller-Hinton agar according to the Clinical and Laboratory Standard Institute (CLSI) guide line [16]. About 3–5 pure colonies of young culture suspension was prepared in equivalent to 0.5McFarland standards and plated. The plates were allowed to dry for 3–5 min; antibiotic discs were evenly distributed on the inoculated plate using sterile forceps and incubated at 37 °C for 18–24 h. The diameter of the zone of inhibition around the antibiotic disc was measured using a ruler. Results were interpreted as Sensitive, Intermediate, and Resistance based on CLSI 2017 guide-line. The antibiotic discs used were: ampicillin (AMP, 10 μg), amoxicillin-clavulanic acid (AMC, 20/10 μg), cotrimoxazole (SXT, 25 μg), tetracycline (TET, 30 μg), ciprofloxacin (CIP, 5 μg), chloramphenicol (CHL, 30 μg), gentamycin (GEN, 10 μg), cefepime (FEP, 30 μg), cefixime (CFM, 5 μg), ceftriaxone (CRO, 30 μg), cefoxitin (FOX, 30 μg), and ceftazidime (CAZ, 30 μg) (all from Abtek bio.Ltd UK) and were selected following CLSI guide-line. Multi-drug resistance patterns of the isolates were determined following the criteria set by Magiorakos et al. [17].

### Detection of extended-spectrum β-lactamase (ESBL)

Following antimicrobial susceptibility testing bacterial isolates showing zones of inhibition diameters ≤ 22 mm to ceftazidime [30 μg] or ≤ 27 mm to cefotaxime [30 μg] were subjected to ESBL production test. Phenotypic confirmation of ESBL production was done by using the double-disk diffusion method; cefotaxime [30 μg] and cefotaxime-clavulanic acid [30/10 μg] or ceftazidime [30 μg] and ceftazidime clavulanic acid [30/10 μg] as previously described [10, 16].

### Detection of carbapenemase producer bacteria

Carbapenemase-producing isolates of Gram-negative bacteria were phenotypically investigated by Modified Hodge Test (MHT). A 0.5 McFarland suspension of carbapenem susceptible strains of *E. coli* ATCC25922 was used as a lawn culture over Mueller Hinton agar plates and meropenem (10 µg) disc was placed at the centre of the plates. With the help of sterile loop, pure colonies of test strains were streaked on the plate from meropenem disk towards the edge. A positive control, *K. pneumoniae* ATCC1705 and negative control, *K. pneumoniae* ATCC1706 were used in the same plate. Inoculated plates were kept for 15 min at room temperature and incubated at 37^0^C for 24 h. Observing a clover leaf like shape was considered as positive for carbapenemase production [10, 16].

### Data analysis

Data were entered and analyzed using SPSS version 20. Descriptive statistics were applied to see the distribution of sociodemographic variables. Frequency and percentages were computed using descriptive statistics. A P-value less than 0.05 at a 95% confidence interval was considered statistically significant.

### Quality control

Five percent of questionnaires were pretested and checked for completeness before the commencement of the actual work. All the laboratory activities were done as per standard operating procedures. The sterility and the performance of the media were checked daily. To check the sterility, 5% of the prepared media were incubated overnight at 37 °C for 24 h. *E*. *coli* (ATCC 25922), *K*. *pneumoniae* (ATCC1705) and *K. pneumoniae* (ATCC1706) were used as quality control for identification and antimicrobial susceptibility testing. The reliability of the findings was guaranteed by implementing quality control measures such as pre-analytical, analytical and post-analytical throughout the whole processes of the laboratory work. Multi-drug resistance was considered as simultaneous resistance to 3 or more antibiotic classes.

## Results

A total of 833 patients were enrolled in the present study. Of these 388 (46.6%) were females and 445 (53.4%) were males. Majority of the study participants were age ≤ 5 years, 252(30.3%) followed by age group 16–30 years, 211(25.3%); 31–45 years, 150 (18.0%) and the least age groups were age greater than 60 years, 39 (4.7%). Four hundred eighty-five (58.2%) were urban residence and 348(41.8%) were rural residence (Table 1).

**Table 1:** Sociodemographic characteristics of patients attending the three Referral Hospitals of Amhara region, Ethiopia; 2017–2018

Sociodemographic characteristics	Frequency	Percentage (%)
Sex		
Male	388	46.6
Female	445	53.4
Age (years)		
≤ 5	252	30.3
6–15	107	12.8
16–30	211	25.3
31–45	150	18.0
46–60	74	8.9
> 60	39	4.7
Residence		
Urban	485	58.2
Rural	348	41.8
Educational status		
Illiterate	103	12.4
Primary	226	27.1
Secondary	128	15.4
Diploma and above	124	14.9
Children ≤ 5 years	252	30.3
Patient setting		
OPDs	417	50.1
Wards	416	49.9
Occupation		
Employed	117	14.0
Merchant	74	8.9
Housewife	81	9.7
Daily labourer	20	2.4
Farmer	56	6.7
Children ≤ 5 years	252	30.3
Others	233	28.0

### Gram-negative bacterial profile in different clinical samples

A total of 833 clinical samples (blood, urine, wound discharges, abscesses, body fluid and stool) were cultured for bacterial growth, and 141 (16.9%) Gram-negative bacteria were identified. Culture positivity was higher in discharges 31 (50.8%); followed by urine culture 66 (41.8%); and abscess 33 (38.4%) (table not presented).

Majority of Gram-negative bacteria were isolated from urine 45/141 (31.9%) followed by blood culture 39/141 (27.7%), wound and abscesses 17/141(12.1%) each, and discharges 15/141 (10.6%). The most common isolates in urine culture were *E. coli* 27/45(60%) followed by *Klebsiella* spp. 12/45 (26.7%), and Citrobacter spp. 4/45(8.9%). The most common isolates in blood culture were *E. coli* and *Klebsiella* spp. 11/39(28.2%) (each), and *Enterobacter* spp. 8/39(20.5%). As *Proteus* spp was a common isolate in wound 6/17(35.3%); *Klebsiella* spp was common in abscesses 8/17 (47.1%) and discharges (Eye, Ear and Nasal) 5/15 (33.3%) each (Table 2).

**Table 2:** Gram-negative bacterial profile in different clinical samples from the three Referral Hospitals of Amhara region, Ethiopia; 2017–2018

Clinical specimen	Isolates	Frequency	Percentage
Blood	*Citrobacter* spp	2	5.1
	*Enterobacter* spp*.*	8	20.5
	*E. coli*	11	28.2
	*Klebsiella* spp.	11	28.2
	*P. aeruginosa*	2	5.1
	*Proteus* sp*.*	1	2.6
	*Salmonella* sp.	1	2.6
	*Providencia* spp*.*	3	7.7
	Total	39	100
Urine	*Citrobacter* spp	4	8.9
	*E. coli*	27	60.0
	*Klebsiella* spp.	12	26.7
	*Proteus* spp.	2	4.4
	Total	45	100
Wound	*Citrobacter* spp	3	17.7
	*Enterobacter* spp*.*	2	11.8
	*E. coli*	1	5.9
	*Klebsiella* spp*.*	2	11.8
	*P. aeruginosa*	2	11.8
	*Proteus* spp.	6	35.3
	*Providencia *sp*.*	1	5.9
	Total	17	100
Discharges	*Citrobacter* spp.	2	13.3
	*Enterobacter* sp*.*	1	6.7
	*E. coli*	2	13.3
	*Klebsiella* spp.	5	33.3
	*P. aeruginosa*	1	6.7
	*Proteus* spp.	3	20.0
	*Moraxilla* sp	1	6.7
	Total	15	100
Abscesses	*Citrobacter* sp	1	5.9
	*Enterobacter* spp*.*	2	11.8
	*E. coli*	2	11.8
	Klebsiella spp.	8	47.1
	*P. aeruginosa*	1	5.9
	*Providencia *spp.	2	11.8
	*Morganella* sp.	1	5.9
	Total	17	100
Body fluids	*E. coli*	3	75
	*Proteus* sp.	1	25
	Total	4	100
Stool	*Salmonella* spp.	2	50
	*Shigella* spp.	2	50
	Total	4	100
Total		141	100%

### Distribution of clinical isolates in the three referral hospitals, Amhara region

**Figure 1:**
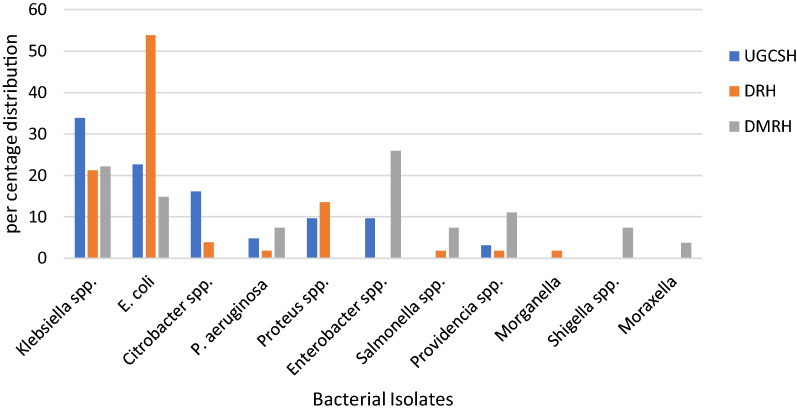
Distribution of bacterial isolates from the three Referral Hospitals of Amhara region, Ethiopia; 2017–2018. (*UGCSH* University of Gondar Comprehensive Specialized Hospital, *DRH* Dessie Referral Hospital, *DMRH* Debre Markos Referral Hospital)

The distribution of clinical isolates in the three referral hospitals demonstrate that 44% were from the University of Gondar Comprehensive Specialized Hospital, 36.9% were from Dessie Referral Hospital and 19.1% were from Debre Markos Referral Hospital. The most common isolates at the University of Gondar Comprehensive Specialized Hospital was *Klebsiella* spp. 33.9% followed by *E. coli* 22.6% and *Citrobacter* spp. 16.1%; in Dessie Referral Hospital *E. coli* 53.8%, *Klebsiella* spp. 21.2% and *Proteus* spp. 13.5%; in Debre Markos Referral Hospital *Enterobacter* spp. 25.9%, *Klebsiella* spp. 22.2% and *E. coli* 14.8%. The overall distribution of the isolates in the three referral hospitals were *E. coli* 32.6%, *Klebsiella* spp. 26.5% and *Proteus* spp. 9.2% (Fig. 1).

### Antibiotic resistance pattern

Among 141 isolates tested for different antibiotics, 137 (97.2%) were resistant to ampicillin followed by cotrimoxazole 115 (81.6%), amoxicillin clavulanic acid 109 (77.3%), cefixime 99 (70.2%), cefepime 93 (66.0%) and tetracycline 91 (64.5%). However, comparatively low rates of resistance were observed for ciprofloxacin 55 (39.0%), chloramphenicol 61 (43.3%) and cefoxitin 65 (46.1%) (Table 3).

**Table 3:** Drug resistance patterns of Gram-negative bacterial isolates against commonly used antibiotics from the three Referral Hospitals of Amhara region, Ethiopia; 2017–2018

Bacterial Isolates		AMP	AMC	SXT	TET	*CIP*	CHL	GEN	FEP	CFM	FOX	CRO	CAZ
*E. coli* (n = 46)	S	2	8	6	11	25	31	24	12	11	29	22	16
	I	–	4	1	4	2	2	3	10	4	5	5	6
	R	44	34	39	31	19	13	19	24	31	12	19	24
*Klebsiella* spp*.* (n = 38)	S	–	7	8	8	23	17	11	2	9	11	6	6
	I	–	1	–	5	4	5	1	6	3	6	7	6
	R	38	30	30	25	11	16	26	30	26	21	25	26
*Proteus* Spp. (n = 13)	S	2	2	1	3	7	6	4	4	2	4	3	2
	I	–	1	–	2	1	–	1	1	2	2	2	4
	R	11	10	12	8	5	7	8	8	9	7	8	7
*Enterobacter* Spp. (n = 13)	S	–	1	2	7	8	5	5	1	2	5	3	2
	I	–	1	–	–	–	–	–	2	1	2	–	1
	R	13	11	11	6	5	8	8	10	10	6	10	10
*Citrobacter* Spp. (n = 12)	S	–	1	3	2	6	3	6	3	2	3	1	4
	I	–	1	1	1	1	–	–	1	1	–	1	2
	R	12	10	8	9	5	9	6	8	9	9	10	6
*P. aeruginosa (n* = *6)*	S	–	–	–	3	3	–	4	–	–	3	–	–
	I	–	2	–	–	1	1	–	1	1	2	–	1
	R	6	4	6	3	2	5	2	5	5	1	6	5
*Providencia *Spp.* (n* = *6)*	S	–	–	–	–	1	4	5	1	–	1	–	–
	I	–	–	–	–	–	–	–	–	1	–	1	1
	R	6	6	6	6	5	2	1	5	5	5	5	5
*Salmonella* Spp. (n = 3)	S	–	–	1	–	1	2	1	1	1	1	1	1
	I	–	–	–	1	–	–	–	–	–	–	–	–
	R	3	3	2	2	2	1	2	2	2	2	2	2
*Others *(*n* = 4)^a^	S	–	3	3	2	3	4	3	3	2	2	3	3
	I	–	–	–	1	–	–	–	–	–	–	–	1
	R	4		1	1	1	–	1	1	2	2	1	–
*Total(n* = *141)*	S	4 (2.8)	22 (15.6)	24 (17.0)	36 (25.5)	77 (54.6)	72 (51.0)	63 (44.7)	27 (19.1)	29 (20.6)	59 (41.8)	39 (27.7)	34 (24.1)
	I	–	10 (7.1)	2 (1.4)	14 (9.9)	9 (6.4)	8 (5.7)	5 (3.5)	21 (14.9)	13 (9.2)	17 (12.1)	16 (11.3)	22 (15.6)
	R	137 (97.2)	109 (77.3)	115 (81.6)	91 (64.5)	55 (39.0)	61 (43.3)	73 (51.8)	93 (66.0)	99 (70.2)	65 (46.1)	86 (61.0)	85 (60.3)

*Amp* ampicillin, *AMC* amoxicillin-clavulanic acid, *SXT* cotrimoxazole, *TET* tetracycline, *CIP* ciprofloxacin, *CHL* chloramphenicol, *GEN* gentamycin, *FEP* cefepime, *CFM* cefixime, *FOX* cefoxitin, *CRO* ceftriaxone, *CAZ* ceftazidime

^a^*Shigella* spp. (n = 2), *Moraxella* sp. (n = 1), *Morganella* sp*.* (n = 1)

### Multidrug resistant isolates

The MDR determination was carried out by considering the classification of antibiotics in the following six antibiotic classes: penicillin and cephalosporin (beta-lactam drugs); fluoroquinolones; sulphonamides; tetracyclines; chloramphenicol; and aminoglycosides. The MDR isolates across the three referral hospitals were 55, 46, 20 from UGCSH, DRH and DMRH, respectively and the overall MDR prevalence was 121/141 (85.8%) (Table 4). Accordingly, all isolates of *P. aeruginosa* and *Providencia* spp. were MDR, 6 (100%) each, followed by *Proteus* spp. 12 (92.3%), *Klebsiella* spp. 34 (89.5%), *E. coli* 39 (84.8%), *Enterobacter* spp. 11 (84.6%) and Citrobacter spp. 10 (83.3%) (Table 5).

**Table 4:** Distribution of MDR isolates from the three Referral Hospitals of Amhara region, Ethiopia, 2017–2018

Isolates	Study sites						Total	
	UGCSH^a^		DRH		DMRH		Non-MDR, N (%)	MDR, N (%)
	Non- MDR, N (%)	MDR, N (%)	Non-MDR N (%)	MDR, N (%)	Non-MDR, N (%)	MDR, N (%)		
*Klebsiella* spp.	2 (3.2)	19 (30.6)	1 (1.9)	10 (19.2)	1 (3.7)	5 (18.5)	4 (10.5)	34 (89.5)
*E. coli*	2 (3.2)	12 (19.4)	4 (7.7)	24 (46.2)	1 (3.7)	3 (11.1)	7 (15.2)	39 (84.8)
*Citrobacter *spp.	2 (3.2)	8 (12.9)	–	2 (3.8)	–	–	2 (16.7)	10 (83.3)
*P. aeruginosa*	–	3 (4.8)	–	1 (1.9)	–	2 (7.4)	–	6 (100)
*Proteus *spp.	–	6 (9.7)	1 (1.9)	6 (11.5)	–	–	1 (7.7)	12 (92.3)
*Enterobacter *spp.	1 (1.6)	5 (8.1)	–	–	1 (3.7)	6 (22.3)	2 (15.4)	11 (84.6)
*Salmonella *spp.	–	–	–	1 (1.9)	1 (3.7)	1 (3.7)	1 (33.3)	2 (66.7)
*Providencia *spp.	–	2 (3.2)	–	1 (1.9)	–	3 (11.1)	–	6 (100)
*Morganella *spp.	–	–	–	1 (1.9)	–	–	–	1 (100)
*Shigella *spp.	–	–	–	–	2 (7.4)	–	2 (100)	–
*Moraxella *sp.	–	–	–	–	1 (3.7)	–	1 (100)	–
Total	7 (11.3)	55 (88.7)	6 (11.5)	46 (88.5)	7 (25.9)	20(74.1)	20 (14.2)	121(85.8)

^a^*UGCSH*  University of Gondar Comprehensive Specialized Hospital, *DRH* Dessie Referral Hospital, *DMRH* Debre Markos Referral Hospital.

**Table 5:** Multidrug resistance profile of Gram-negative bacteria isolated from clinical samples at the three Referral Hospitals of Amhara region, Ethiopia; 2017–2018

Antibiogram pattern	Bacterial isolates									Total, N = 141 (%)
	*E. coli*	*Klebsiella* spp*.*	*Proteus *spp.	*Enterobacter* spp.	*Citrobacter* spp.	*P. aeruginosa*	*Providencia *spp.	*Salmonella* spp	Others^a^	
All drug sensitive	1									1
AMP (Not MDR)	1	1							2	4
AMP, AUG (Not MDR)	1							1		2
AMP, SXT (Not MDR)				1						1
AMP, GEN (Not MDR)		1								1
AMP, AUG, SXT (Not MDR)	1		1							2
AMP, FEP, FOX (Not MDR)	1									1
AMP, AUG, TET, FOX (Not MDR)		1								1
AMP, AUG, FEP, FOX (Not MDR)		1								1
AMP, AUG, CHL, FEP (Not MDR)					1					1
AMP, TET, CRO, FOX (Not MDR)					1					1
AMP, AUG, SXT, FOX (Not MDR)				1						1
AMP, FEP, CFM, FOX (Not MDR)									1	1
AMP, AUG, SXT, FOX, CAZ (Not MDR)	1									1
AMP, AUG, SXT, CFM, CAZ (Not MDR)	1									1
AMP, SXT, TET (MDR)	2	1								3
AMP, CHL, GEN (MDR)	1									1
SXT, TET, CRO (MDR)			1							1
Resistant to 4–12 antibiotics (others MDR)	36	33	11	11	10	6	6	2	1	116
Total Non MDR isolates	7 (15.2)	4(10.5)	1(7.7)	2(15.4)	2 (16.7)	–	–	1(33.3)	3(75)	20 (14.2)
Total MDR-isolates	39 (84.8)	34 (89.5)	12(92.3)	11 (84.6)	10 (83.3)	6(100)	6(100)	2 (66.7)	1(25.0)	121 (85.8)
Total (MDR + Non-MDR)	46 (100)	38 (100)	13 (100)	13 (100)	12 (100)	6 (100)	6 (100)	3 (100)	4 (100)	141 (100)

*Amp* ampicillin, *AMC* amoxicillin-clavulanic acid, *SXT* cotrimoxazole, *TET* tetracycline, *CIP* ciprofloxacin, *CHL* chloramphenicol, *GEN* gentamycin, *FEP* cefepime, *CFM* cefixime, *FOX* cefoxitin, *CRO* ceftriaxone, *CAZ* ceftazidime

MDR = isolates resistant to 3 or more antibiotics classes (The antibiotic classes used in this table are Penicillin and Cephalosporins (beta-lactam drugs), Fluoroquinolones, Sulphonamides, Tetracyclines, Chloramphenicol, Aminoglycosides)

*Others *^a^ *Morganella *sp.* (n* = *1), Shigella *spp.* (n* = *2), and Moraxella *sp.* (n* = *1)*

### ESBL and carbapenemase-producing isolates

Of 141 isolates, 124 isolates of Gram-negative bacteria were tested phenotypically for ESBL production. Of these, 59 were from the University of Gondar Comprehensive Specialized Hospital and 47 were from Dessie Referral Hospital and 18 were from Debre Markos Referral Hospital; and 25.8%, 19.4%, 10.5% were ESBL positive respectively. The most common ESBL producing isolates at the University of Gondar Comprehensive Specialized Hospital was *Klebsiella* spp. 9.7% and *E. coli* 5.7%, at Dessie Referral Hospital, *E. coli* 10 (8.1%) and *Klebsiella* spp. 5 (4%) and at Debre Markos Referral Hospital *Enterobacter* spp. 5 (4.0%) were isolated. Of the total isolates the most common ESBL producing isolates were *Klebsiella* spp. 19 (15.3%) and *E. coli* 17 (13.7%). The overall prevalence of ESBL producing GNB was 69 (55.6%) (Table 6).

**Table 6:** Distribution of ESBL and carbapenemase producing isolates from the three Referral Hospitals of Amhara region, Ethiopia; 2017–2018

Hospitals	Isolates	ESBL N = 124 (%)			MHT N = 51 (%)		
		Positive	Negative	Total	Positive	Negative	Total
University of Gondar Comprehensive Specialized Hospital	*Klebsiella *spp.	12 (9.7)	8 (6.5)	20 (16.1)	2 (3.9)	9 (17.7)	11 (21.6)
	*E. coli*	7 (5.7)	6 (4.8)	13 (10.5)	–	7 (13.7)	7 (13.7)
	*Citrobacter spp*	4 (3.2)	6 (4.8)	10 (8.1)	1 (2.0)	2 (3.9)	3 (5.9)–
	*P. aeruginosa*	1 (0.8)	2 (1.6)	3 (3.4)	–	–	–
	*Proteus *spp.	4 (3.2)	1 (0.8)	5 (4.0)	–	2 (3.9)	2 (3.9)
	*Enterobacter *spp.	3 (3.4)	3 (3.4)	6 (4.8)	–	1 (2.0)	1 (2.0)
	*Providencia *spp.	1 (0.8)	1 (0.8)	2 (1.6)	–	–	–
	Total	32 (25.8)	27 (21.8)	59 (47.6)	3 (5.9)	21 (41.2)	24 (41.1)
Dessie Referral Hospital	*Klebsiella *spp.	5 (4.0)	4 (3.2)	9 (7.3)	–	3 (5.9)	3 (5.9)
	*E. coli*	10 (8.1)	15 (12.1)	25 (20.2)	1 (2.0)	10 (19.6)	11 (21.6)
	*Citrobacter spp*	2 (1.6)	–	2 (1.6)	–	2 (3.9)	2 (3.9)
	*P. aeruginosa*	–	1 (0.8)	1 (0.8)	–	–	–
	*Proteus *spp.	4 (3.2)	3 (3.4)	7 (5.6)	–	3 (5.9)	3 (5.9)
	*Salmonella *spp.	1 (0.8)	–	1 (0.8)	–	–	–
	*Morganella spp*	1 (0.8)	–	1 (0.8)	–	–	–
	*Providencia *spp.	1 (0.8)	–	1 (0.8)	–	–	–
	Total	24 (19.4)	23 (18.5)	47 (37.9)	1 (2.0)	18 (35.3)	19 (37.3)
Debre Markos Referral Hospital	*Klebsiella *spp.	2 (1.6)	3 (3.4)	5 (4.0)	1 (2.0)	1 (2.0)	2 (3.9)
	*E. coli*	–	1 (0.8)	1 (0.8)	–	–	–
	*P. aeruginosa*	2 (1.6)	–	2 (1.6)	–	–	–
	*Enterobacter *spp.	5 (4.0)	1 (0.8)	6 (4.8)	3 (5.9)	2 (3.9)	5 (9.8)
	*Salmonella *spp.	1 (0.8)	–	1 (0.8)	–	1 (2.0)	1 (2.0)
	*Providencia *spp.	3 (3.4)	–	3 (3.4)	–	–	–
	Total	13 (10.5)	5 (4.0)	18 (14.5)	4 (7.8)	4 (7.8)	8 (15.7)
Total		69 (55.6)	55 (44.4)	124 (100)	8 (15.7)	43 (84.3)	51 (100)

Among 124 isolates tested for ESBL; 51 MDR isolates were further checked for carbapenemase production. Of these isolates, proportionally 24 isolates were selected from the University of Gondar Comprehensive Specialized Hospital, 19 from Dessie Referral Hospital and 8 from Debre Markos Referral Hospital. Of these isolates 5.9%, 2.0% and 7.8% were positive for carbapenemase respectively. *Enterobacter*, *Klebsiella* and *E. coli* were reported positive for MHT. The overall prevalence of carbapenemase producing GNB was 8 (15.7%) (Table 6).

## Discussion

The overall prevalence of Gram-negative bacteria isolated from various clinical samples (blood, urine, wound discharges, abscesses, body fluid and stool) from patients attending the three referral hospitals was 16.9%. This is similar in studies from Mexico, where 19.1% of the isolates were Gram-negative bacteria [18], and Nepal, 17% [19]. However, it is lower than a study from Bahir Dar / Ethiopia, 34.8% [10]. The most common isolates in the three Referral Hospitals were *E. coli,* 32.6% and *Klebsiella* spp., 26.5%. This agrees with results of a Saudi Arabian study that revealed the same results in which, *E. coli,* 69.8% and K. *pneumoniae,* 17.2% were the most frequently isolated Gram-negative bacteria [20]. Another study from Ethiopia, reported that the most common isolates were *K. pneumoniae,* 52.4% and *E. coli*, 12.4% [10], which is in congruent with findings of the current study. But a study in Iran showed that the most frequently isolated bacteria were *Enterobacter aerogenes*, 50.6% followed by *E. coli,* 16.7% and *Pseudomonas aeruginosa*, 7.5% [21]. The sources and numbers of the clinical samples collected, type of infections, patient types or wards at which the samples obtained, and geographical differences used in each study may explain the observed variations among the overall prevalence and occurrences of Gram-negative bacteria.

Regarding antimicrobial resistance rate of GNB a high resistance rate was observed to the commonly prescribed antimicrobials, except for ciprofloxacin and chloramphenicol to which the isolates exhibited below 50% resistant. This result is similar to reports from Iran, where the resistant patterns of GNB to ciprofloxacin was 36% [21]. Similarly, a Mexican report showed that the isolated GNB exhibited a high resistance rate for ampicillin (95.85%), cefuroxime (84.17%), piperacillin (82.93%), cefotaxime (78.07%), ceftriaxone (77.41%), aztreonam (75.23%), cefazolin (75.00%), and ceftazidime (73.19%) [18]. In a study from Nepal, *E. coli* was found to be most sensitive to cephalosporins and tetracycline and most resistant to quinolones, fluroquinolones and sulphonamides [22]. These high rates of antimicrobial resistance observed in our setting, alarms the stakeholders to have more surveillance and control of the use of antimicrobials to combat infections.

The overall prevalence of MDR GNB in this study was 85.8% (95% CI:80.04%, 91.5%) which is marginally in line with a study conducted at Bahir Dar/Ethiopia where the MDR GNB was 80% [10]. A study from Nepal also revealed that MDR GNB was 82.5% [19]. *P. aeruginosa* and *Providencia* spp. were 100% MDR GNB in the present study; however, the most common MDR isolates at Bahir Dar/Ethiopia were *Klebsiella pneumoniae*, 87.6% followed by *E. aerogenes*, 83.3%, *E. coli*, 82.6%, *E. cloacae*, 77.8% [10]. A report from Mexico also showed that the highest percentage of MDR profile was observed in *E, coli*, 91.57%, and *Acinetobacter baumannii,* 86.79% [18]. In Iran, MDR GNB were documented in 25.8% of *Acinetobacter* spp., 20% of *Klebsiella* spp., and 16.6% of *Pseudomonas* spp. The most active antimicrobials were vancomycin, 93.5% followed by amikacin, 71.5% and gentamicin, 46% [21], whereas relatively active antibiotics in the present study were ciprofloxacin, chloramphenicol, and cefoxitin. Relatively lower resistance to cefoxitin in the current study is probably associated with the lower clinical risk factors of the study participants for cefoxitin resistance. For instance, patients with none of the risk factors such as hospitalization and antimicrobials usage within a month before data collection, nursing home residency before admission, bladder surgical diversion or long-term catheterization are less likely to have cefoxitin resistance [23]. In addition, cefoxitin has a parenteral route and thus not as such accessible to the community in the study setting unlike to other common antimicrobials which have an oral route.

The global prevalence of ESBL producing Gram-negative bacteria (GNB) varies, and the higher prevalence rates were from the developing continents such as South America, Asia [24], and Africa [25]. The prevalence of ESBL found in this study was 55.6% which is the same as from studies in Nepal, 55.6% (19); Ethiopia/Bahir Dar, 57.8%, [26], Cameroon, 55.3% [27]; and India, 54.3% [28]. However, it is higher than studies conducted in Jimma/Ethiopia, 36% [29]; and Gondar/ Ethiopia, 18.2% [30]; and Nigeria (20.9%) [31]; Pakistan (44.3%) [32]; and Chad (47.7%) [33]. However, the current result is lower than the studies from Bahir Dar/Ethiopia 85.8% [10] and Jimma/Ethiopia, 63.4% (71/112) [34]. The most common ESBL producing isolates were *E. coli* 43.6% and *Klebsiella*spp.55.9%. This is lower than a study from Mexico, a majority of ESBL-producing isolates was *E. coli* 83.13%, *Klebsiella pneumoniae* 78.84% [18]; and from Nepal, *E. coli* 70.9% and *Klebsiella* spp. 59.4% were common ESBL producing isolates reported [35]. A possible explanation of the observed variation across various studies conducted globally could be the types of patients involved in the study, for instance, the study participants might be outpatients, inpatients, intensive care unit (ICU) patients, patients with different underlined diseases, and these characteristics of patients in the hospitals allow for considerable factors that lead to antimicrobial resistance. These factors may include everyday use of broad-spectrum antimicrobial drugs, use of invasive procedures and devices, patients with a high frequency of comorbidity, and prolonged hospital stays, among others [36].

Of the 51 isolates screened for carbapenemase production by using the Modified Hodge Test (MHT), 8 (15.7%) were positive. This is in line with the studies from Bahir Dar/Ethiopia, 16.2% [10]. But it is higher than studies from Ghana, 2.9% [37]; China, 1% [38]; and Germany, 1.2% [39]. However, the present result is lower than reports from Sudan, 50% [40]; Uganda, 22.4% [41]; and Yemen, 25.3% [42]**.** These observed variations may be due to restricted use of antibiotics in those developed countries compared to the developing countries where most drugs are available over the counter without prescription by a clinician [43, 44]. For instance, the pooled estimate of inappropriate antibiotic use in Ethiopia was 49.2%, and the pooled proportion of self-antibiotic prescription was, 43.3% [43]. Other reasons for inappropriate use of antibiotics may include a wrong indication, wrong duration, improper route of administration, use of leftover antibiotics from a family member, and immature discontinuation of antibiotics.

## Conclusion and recommendation

Multidrug-resistance and ESBL producing isolates in the present study were high. *E. coli* and *Klebsiella* spp*.* were the most common ESBL producing GNB. *Klebsiella* spp*., Enterobacter* spp*., E. coli* and *Citrobacter* spp*.* were carbapenemase- producing isolates. Continuous monitoring, antibiotic stewardship and molecular detection of the gene responsible for drug resistance are important means to reduce the spread of drug- resistant pathogens.

## Data Availability

All data generated or analysed during this study were included in this article.

## Abbreviations

MDR: Multidrug resistance
ESBL: Extended-spectrum beta-lactamase
CLSI: Clinical and Laboratory Standard Institute
MHT: Modified Hodge Test
GNB: Gram-negative bacteria
